# Change in child mental health during the Ukraine war: evidence from a large sample of parents

**DOI:** 10.1007/s00787-023-02255-z

**Published:** 2023-07-08

**Authors:** Eoin McElroy, Philip Hyland, Mark Shevlin, Thanos Karatzias, Frédérique Vallières, Menachem Ben-Ezra, Maria Louison Vang, Boris Lorberg, Dmytro Martsenkovskyi

**Affiliations:** 1 School of Psychology, Ulster University, Derry, Northern Ireland, UK; 2 Department of Psychology, Maynooth University, Kildare, Ireland; 3 School of Health and Social Care, Edinburgh Napier University, Edinburgh, Scotland, UK; 4 Trinity Centre for Global Health, Trinity College Dublin, Dublin, Ireland; 5 School of Social Work, Ariel University, Ariel, Israel; 6 Centre for Psychotraumatology, University of Southern Denmark, Odense, Denmark; 7 Department of Psychiatry, University of Massachusetts Chan Medical School, Worcester, MA USA; 8 Department of Psychiatry and Narcology, Bogomolets National Medical University, Kyiv, Ukraine; 9 Institute of Psychiatry, Forensic Psychiatric Examination and Drug Monitoring, Ministry of Health of Ukraine, Kyiv, Ukraine

**Keywords:** Ukraine war, Internalizing, Externalizing, Attention, War trauma

## Abstract

The ongoing war in Ukraine is expected to negatively impact the mental health of the country’s population. This study aims to provide a preliminary estimate of the degree of change in the mental health problems of Ukrainian children following Russia’s invasion in February 2022, and to identify the sociodemographic and war-related risk factors associated with these changes. A nationwide, opportunistic sample of 1238 parents reported on a single randomly chosen child within their household as part of The Mental Health of Parents and Children in Ukraine Study. Data were collected between July 15th and September 5th, 2022. Participants completed modified versions of the Pediatric Symptom Checklist (PSC-17) which was adapted to capture change in the frequency of symptoms since the beginning of the war. Parents reported increases across all 17 indicators of internalizing, externalizing, and attention problems of the PSC-17. Increased problems were most pronounced within the internalizing domain, with 35% of parents reporting that their child worried more since the beginning of the war. A number of individual, parental, and war-related factors were associated with increases across the three domains. Exposure to war trauma, pre-existing mental health problems, and child age were among the strongest predictors of change. This survey provides preliminary evidence that the Russian war on Ukraine has led to an increase in common mental health problems among children in the general population. Further research is required to determine the extent and sequela of this increase, and to develop intervention strategies for those most in need.

**Supplementary Information:**

The online version contains supplementary material available at 10.1007/s00787-023-02255-z.

## Introduction

The invasion of Ukraine by Russia in February 2022 has brought about humanitarian and economic crises on a scale not seen in Europe since the Second World War. As of mid-November 2022, the Office of the United Nations High Commissioner for Human Rights (OHCHR) has corroborated over 16,600 civilian casualties, including over 6550 deaths, of which over 400 were children [1]. To date, more than half of Ukraine’s 7.5 million children have been displaced by the war, of which 2.8 million are internally displaced [2]. The long-term economic and societal damage inflicted to date is estimated to cost $350 billion in order to restore key infrastructure, including damages caused to roads, hospitals, schools, and water treatment facilities [3]. As is the case with all wars, children have not been spared the horrors. In addition to those confirmed to have been killed or seriously injured, millions more children have experienced disruptions to their homes, education, family units, and access to basic health and sanitation services [2]. Accordingly, researchers have predicted increased mental health problems in the general population of Ukraine [4], particularly among young people [5, 6], and UNICEF anticipates that over 2.2 million children are in need of accessing mental health and psychosocial support [2].

There is an abundance of literature linking exposure to war with various forms of mental health problems in children and adolescents. Several reviews have identified a robust association between war exposure and both internalizing (e.g. depression and anxiety) and externalizing problems (e.g. disruptive behaviour) [7–9]. Elevated risk for attention-deficit/hyperactivity disorders (ADHD) has also been reported for war-exposed children [10]. Although the ongoing conflict has made the gathering of representative data difficult, preliminary evidence of the mental health impact of this war is beginning to emerge. A May 2022 survey of 589 university students and staff members living in Ukraine reported considerable increases in psychological distress, burnout, loneliness, and substance use since the beginning of the war [11]. Studies carried out by our research team using a nationwide, opportunistic sample (*N* = 2004) of adult Ukrainians have found that 15% met criteria for a diagnosis of posttraumatic stress disorder (PTSD) and a further 26% met criteria for a diagnosis of Complex PTSD (CPTSD) [12], along with increased symptoms of anxiety, depression, loneliness, and hazardous alcohol use since the beginning of the war [13]. Although these findings are not based on representative data, similar deteriorations in the mental health of the general population are highly plausible, and thus require further research, particularly in younger populations.

Although the negative impact of war on mental health is well-established, these effects are not distributed evenly across the population. Indeed, the psychological impact of war is complex and may be influenced by a range of factors including the severity and chronicity of exposure, family structure, social support, socio-economic status, and broader socio-environmental contexts [14]. For children, developmental stage also plays an important role, with different developmental periods characterized by unique responses to war trauma [9]. It is, therefore, important to understand the impact of war on child mental health within the context of a wide variety of individual, familial, and broader social factors. Moreover, by identifying the children who are most at risk for negative mental health consequences of conflict, we can better direct the limited resources to those most in need of help.

This study, therefore, aims to (i) provide a preliminary estimate of the degree of change in parent-reported child mental health problems following Russia’s invasion of Ukraine in February 2022, and (ii) identify the sociodemographic and war-related risk factors associated with these changes. To achieve these objectives, we used data from the ‘The Mental Health of Parents and Children in Ukraine Study’, a nationwide survey of parents living in Ukraine, to model changes in parent-reported children’s internalizing, externalizing, and attention problems, and identify predictors of change for each of these three domains.

## Methods

### Participants and procedures

The Mental Health of Parents and Children in Ukraine Study was designed to understand the impact of Russia’s war on the mental health and day-to-day lives of adults and children living in Ukraine. Inclusion criteria for this study were being aged 18 years or older, being a parent of a child under the age of 18 years, currently living in Ukraine, and capable of completing the survey in Ukrainian. Data were collected by TGM Research between July 15th and September 5th, 2022. TGM Research maintains nationally representative survey panels in 130 countries, including Ukraine. Given the ongoing conflict and mass displacement of people in Ukraine, the collection of a nationally representative sample was not possible; therefore, we used opportunistic sampling methods to recruit participants. However, we took steps to recruit a sample that was as representative as possible—we recruited participants based on combined gender and age-group quotas that reflected the general Ukrainian population. Attempts were also made to include participants from all 24 administrative regions (i.e. oblasts) within the country; however, oblasts within the Eastern part of the country were underrepresented due to their proximity to the frontline. Potential participants were contacted by TGM Research via email, in-app notification, or text message, and were provided with information about the nature of the study. Consenting participants completed the survey online and were remunerated for their time by the survey company. Ethical approval for the study was provided by SI “Institute of Psychiatry, Forensic Psychiatric Examination and Drug Monitoring of Ministry of Health of Ukraine”, Kyiv, Ukraine. A total of 1238 parents provided complete data on both themselves and one child within the household (the child whose birthday was next). Sample characteristics are presented in Table 1.

**Table 1:** Sociodemographic characteristics of the sample (*N* = 1238)

	% or Mean	N or SD
Child sex		
Female	49.19	609
Male	50.80	629
Child age	9.96	3.92
Number of children in house	1.50	0.69
Child with delayed milestone development	11.3	140
Child with emotional or behavioural problems	12.76	158
Current living location in Ukraine		
Western Ukraine	24.72	306
Northern Ukraine	27.79	344
Central Ukraine	19.06	236
Eastern Ukraine	5.01	62
Southern Ukraine	23.42	290
Residential area		
Urban area	73.51	910
Rural area	26.49	328
Property type		
Apartment/house	96.37	1193
Other (including emergency accommodation)	3.63	45
Forced to move to another part of Ukraine	28.43	352
Forced to move to another country	8.48	105
Marital status of parent		
Married or living with partner	78.43	971
Other	21.57	267
Highest education level of parent		
Undergraduate degree or above	61.79	765
No degree	38.21	473
Employment status of parent		
Full-time employed	38.45	476
Other	61.55	762
Parent war-related stressors	9.02	4.31
Parent PTSD	10.48	4.79
Parent CPTSD	17.44	8.73
Parent change in depression	12.10	7.67
Parent change in anxiety	11.40	6.86
Study-child exposure to war	53.80	666

### Measures

#### Changes in internalizing, externalizing, and attention problems

Changes in internalizing, externalizing, and attention problems were measured using a modified version of the parent report Pediatric Symptom Checklist (PSC-17) [15]. The PSC-17 is a widely used screening measure for child and adolescent mental health problems, and it has previously been used in the Ukrainian population [16]. It consists of three subscales, with five items capturing internalizing, seven questions measuring externalizing, and five items tapping attention problems. Responses are typically indicated on a 3-point Likert scale (‘Never’; ‘Sometimes’; ‘Often’), however, to capture change in the symptoms since the onset of the war, the wording of the instructions and the Likert scale response options were altered. Participants were presented with the following instructions: “Below are a list of feelings and behaviours. Please indicate if your child has been showing signs of each one less often, about the same, or more often since the war began. Just make one choice for each row.” The Likert response scale was changed to reflect “Less Often”, “About the Same”, and “More Often”. For the present analyses, the responses to the PSC-17 items were recoded as 0 (“Less Often” or “About the Same”) or 1 (“More Often”), to ensure that composite scores were unidirectional (i.e. reflecting an increase in mental health problems). The PSC-17 was translated in line with the World Health Organisation’s guidance [17] from English into Ukrainian and then back-translated from Ukrainian into English to ensure accuracy by a team of mental health experts fluent in both languages and familiar with the measures. Everyone involved in the translation process was Ukrainian and educated to at least doctoral level, including a consultant child psychiatrist.

### Analytic plan

The reported increases in the 17 mental health symptoms (expressed as percentages) were visualized using ggplot2 [18]. The binary indicators were summed to form total scores, and descriptive statistics were produced to capture the average increase in internalizing, externalizing, and attention problems at the construct level.

We used categorical confirmatory factor analysis to examine the latent structure and psychometric properties of our modified version of the PSC-17. A three-factor model was specified (increase in internalizing, externalizing, and attention problems), and model fit was determined using the following indices: the Chi-square statistic, the comparative fit index (CFI) [19], the Tucker–Lewis Index (TLI) [20], and the Root Mean Square Error of Approximation (RMSEA) [21]. CFI and TLI values of greater than 0.90 indicate acceptable model fit [22]. General guidelines suggest that RMSEA values of less than 0.05 indicate close fit and values up to 0.08 indicate reasonable errors of approximation [23]. The precision of measurement of the three factors was assessed by plotting total information functions for the three factors (Fig. S1).

In order to explore the associations between our range of covariates and the increase in mental health problems, we employed a structural equation modelling approach (SEM). Latent (increase in) internalizing, externalizing, and attention factors were regressed on the following predictor variables: sex (0 = females, 1 = males)*,* age of the child in years (standardized)*,* number of children in the household (standardized)*,* whether the study child has received treatment for an emotional or behavioural problem (0 = no, 1 = yes)*,* parent report of a developmental problem, such as a delay in speech development or walking without support (0 = no, 1 = yes), parent marital status (0 = married or living with a partner, 1 = other), parent educational level (0 = completed a university degree, 1 = less than degree-level education), parent employment status (0 = full-time employed, 1 = other)*,* current living location in Ukraine (dummy coded variables representing living in northern Ukraine, central Ukraine, eastern Ukraine, and southern Ukraine—reference category is western Ukraine), residential area (0 = rural, 1 = urban)*,* current property type (0 = living in a house or apartment, 1 = other including emergency accommodation), parent war-related stressors (a list of 35 unique war experiences; summed and standardized), forced relocation within Ukraine (0 = no; 1 = yes), and forced relocation outside of Ukraine (0 = no; 1 = yes). Parental PTSD and CPTSD symptoms were also included as covariates and were measured using the International Trauma Questionnaire [24] (scores summed then standardized), along with parental changes in depression and anxiety since the beginning of the war, measured using modified versions of the PHQ-9 [25] and GAD-7 [26] (summed and standardized)*.* Study-child exposure to the war was also included, as measured by a single question: “Has your child been exposed, directly or indirectly, to any event during the war that he or she has found extremely scary?” (0 = no, 1 = yes). The measures of parental war trauma and mental health are described in full detail in the online supplement (Methods S1), and complete survey documentation can be downloaded from the open science framework (https://osf.io/rja3p/). All models were estimated in Mplus version 8.3 [27] using the weighted least squares estimator (WLSMV) estimator.

## Results

**Fig. 1:**
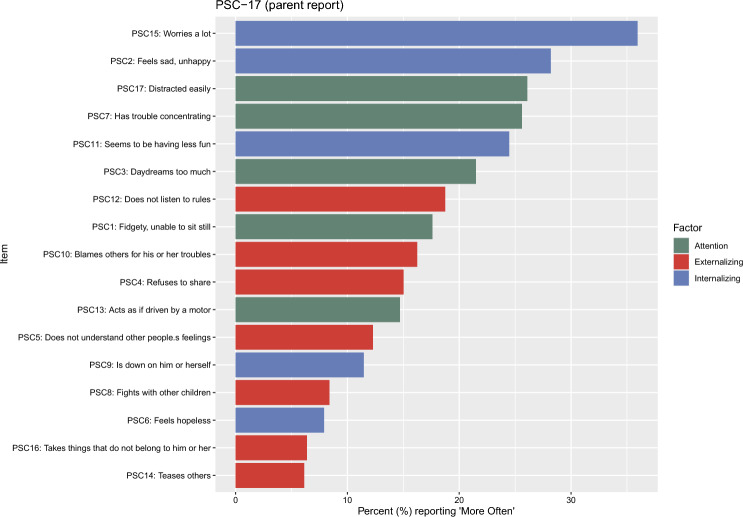
Change in parent reports of internalizing, externalizing and attention problems. Presented as % of parents who endorsed symptoms occurring ‘more often’ since the beginning of the war. See Table S1 for original 3-point Likert scoring

Changes in parent-reported internalizing, externalizing, and attention problems are presented in Fig. 1. The largest increase was for the PSC item “Worries a lot”, with 35.9% of parents reporting that their children experienced this problem more often since the beginning of the war. The increases were generally more pronounced for internalizing and attention indicators compared with externalizing problems. The mean number of internalizing symptoms reported to have increased since the start of the war was 1.08 (SD = 1.38; range = 0–5). For externalizing symptoms, the number was 0.83 (SD = 1.38; range = 0–7), and for attention problems, the increase was 1.06 (SD = 1.35; range = 0–5).

**Fig. 2:**
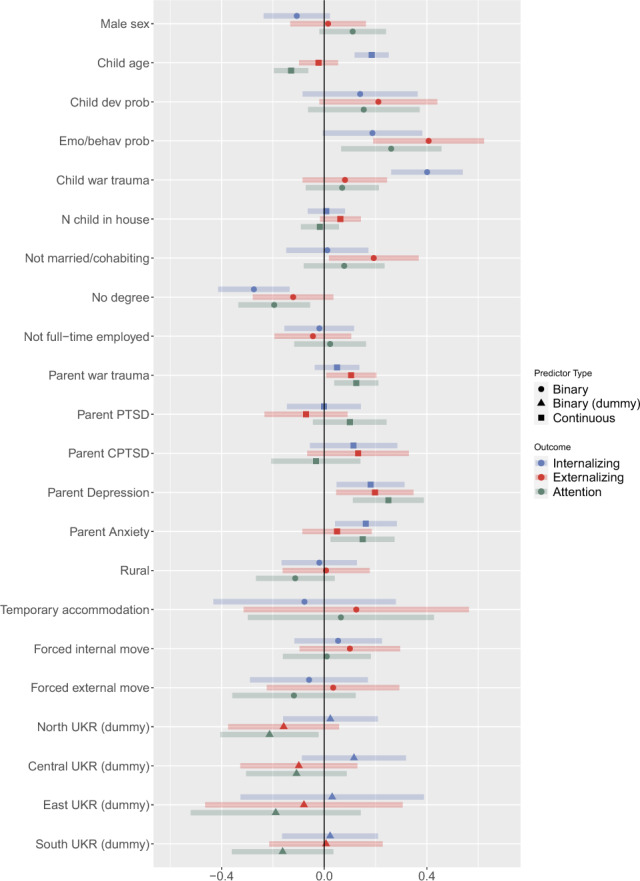
Standardized regression coefficients (with 95% confidence intervals) from the SEM model. Outcomes are latent variables reflecting increases in internalizing, externalizing and attention variables since the start of the war. Predictors are either binary variables, standardized continuous variables, or dummy coded nominal variables (for current location)

Tetrachoric correlation coefficients of the increases in PSC items are presented in the online supplement (Fig. S2). The correlations were generally stronger within (rather than across) the three subscales. Model fit statistics for both the CFA and SEM are presented in Table 2. Both models provided a good fit to the data. Standardized factor loadings from the CFA model are presented in the online supplement (Table S1). The correlations between the latent factors were: *r* = 0.67 for internalizing and externalizing, *r* = 0.69 for internalizing and attention, and *r* = 0.79 for externalizing and attention. The model explained 34.6% of the variance in the change of internalizing problems, 31.3% of the change in attention problems, and 21.3% of the variance in the change of externalizing. Standardized regression coefficients from the SEM are presented in Fig. 2.

**Table 2:** Model fit for CFA and SEM

Model	*χ*^2^	*df*	CFI	TLI	RMSEA
CFA	393.259	116	0.959	0.952	0.044
SEM	714.835	424	0.942	0.93	0.024

Higher internalizing problems were positively associated with the age of the child, child exposure to war trauma, and parental depression and anxiety. Increased internalizing problems were negatively associated with parental education (not having a degree or above). Increased externalizing problems were positively associated with the child having prior emotional and/behavioural problems, living in a single-parent household, parental exposure to war trauma, and parental depression. Increased attention problems were positively associated with the child having prior emotional and/behavioural problems, parental exposure to war trauma, and parental depression and anxiety. Finally, increased attention problems were negatively associated with parental education (not having a degree or above), residing in North Ukraine (compared to West Ukraine), and the age of the child.

## Discussion

The present study aimed to provide a preliminary estimate of the degree of change in child mental health problems following Russia’s invasion of Ukraine in February 2022. We found evidence of a general increase in parent-reported internalizing, externalizing and attention problems in their children. Internalizing symptoms saw the largest increase, with more than 35% of parents reporting higher levels of worry, and over 25% reporting higher levels of sadness/unhappiness. Approximately, 25% of parents stated that their children were more easily distracted or had more problems with concentration. Although changes in behavioural problems were comparatively less pronounced, between 6 and 18% of parents endorsed increases across seven distinct indicators. These trends are broadly in line with recent studies conducted with adult Ukrainian populations [11, 13]. Although the size of the increases observed in the present study could not be considered large, they offer some of the first empirical evidence that the ongoing war in Ukraine is having a detrimental impact on the mental health of the nation’s children. These increases in psychological care demands may pose a significant challenge for the national paediatric mental health system given its lack of human resources for mental health and destroyed infrastructure [28]. Further studies are urgently required to ensure the mental health consequences for Ukrainian children are fully understood, and strategies developed to help those in need.

Our second aim was to explore how increases across the three mental health domains were associated with a range of individual, family and war-related factors, as identified in previous studies [9, 29]. Child exposure to a war-related event (either direct or indirect) was positively associated with increased internalizing problems (increasing risk by 0.4 of a standard deviation), but was not associated with increased behavioural or attention problems. This is consistent with existing evidence that one of the most reliable predictors of psychological distress in war-affected populations is exposure to war-related stressors or traumas [9, 30]. Previous studies have noted a clear dose–response relationship in this association [9]—however, we were unable to test this given our single-item measure of child war exposure. As such, more in-depth research is urgently needed to establish child mental health in Ukraine is being impacted by both direct and indirect war experiences.

While child exposure to war trauma was positively associated with increased internalizing problems, parent war trauma was associated with increased reports of behavioural and attention problems in the study children, albeit with small effect sizes. This could be interpreted as consistent with the family stress model (FSM) [31] wherein parent-stress can lead to child and adolescent behavioural problems indirectly (e.g. parent trauma → parent psychological distress → difficulties in the parent–child relationship → child mental health difficulties). Other findings in the present study could also be seen to fit within this framework. For instance, we found that single parents, who typically experience greater levels of parental stress and burden [32], reported greater increases in behavioural problems in their children (0.2 SD), which also suggests that the impact of the war on child mental health may have been influenced by the broader family environment. Moreover, increased psychological distress in parents, a key mediator in the FSM [31], was associated with child mental health problems in our data. Although the precise mechanisms by which these variables interact cannot be ascertained in our cross-sectional data, the broad pattern of effects suggests that war-related traumas can impact child mental health via the family system [33]. Accordingly, mental health and psychosocial (MHPSS) programming focussed on family systemic psychosocial support, delivered through child protection responses, and consistent with the second layer of intervention for MHPSS in humanitarian emergencies [34], is recommended as part of the MHPSS support response in Ukraine.

Additional child-specific factors were also associated with greater risks for increased mental health difficulties. Children who were 1 SD above the mean age had a 0.2 SD increase in parent-reported internalizing problems. Conversely, parents of younger children were more likely to report an increase in attention problems (0.15 SD). These trends are broadly in line with findings that the emotional and behavioural consequence of war trauma can vary by age, due to developmental differences in comprehension and attribution [9]. Parent reports of study children having had pre-existing emotional or behavioural problems were also associated with increases across all three mental health domains, which is consistent with research suggesting that the psychological impact of traumatic events can be more pronounced for those with pre-existing mental health problems [35].

Other findings are harder to explain. For instance, we observed that parents who had less than degree-level education reported less deterioration in child internalizing and attention problems. Speculatively, this could be a result of lower health literacy among the parents and as a result of their ability to monitor and understand the covert changes of their children’s behaviour [36]. In addition, children who were resident in North Ukraine had less parent report deterioration in attention problems compared with those in Western Ukraine (which served as the reference category). Although the west of Ukraine has experienced fewer direct attacks than other regions of the country, millions of Ukrainian adults and children have resettled in this region from the east and south of Ukraine. It is possible that the higher levels of parent-reported attention problems in their children was, at least in part, influenced by parents who were forced to move to this region at some point after February 24th, 2022.

Furthermore, it is worth noting that a considerable number of parents reported improvements in their children’ symptoms after the onset of the war (Table S1), and there may be both substantive and methodological explanations for this. First, several of the symptoms assessed may be highly context-specific. For instance, 29% of parents reported that their children fought with their peers “less often” since the beginning of the war. Reduced social contact following the invasion (e.g. school closures) may have led to a reduction in opportunity for children to fight with one another. In addition, the scoring of the PSC must be addressed. This instrument was scored as “Less Often”, “About the Same”, and “More Often” in the present study, therefore, capturing both improvements and deterioration in mental health. However, this was part of a larger battery of questionnaires, including assessment of parental mental health, all of which captured deterioration only. As such, participants may have been primed to interpret the Likert scale of the PSC-17 as a unidirectional scale measuring deterioration only. As it is not possible to determine whether context or method factors were driving the reported improvements in the present data, we focussed our analysis and discussion on deterioration of mental health only.

### Limitations

The findings of the present study should be considered in light of several additional limitations. Given the continually changing situation in Ukraine, including ongoing mass displacement and mounting damage to infrastructure, gathering representative population data is logistically challenging. As such, our sample was not selected using random probability sampling, and therefore, results may not be entirely generalizable. However, we attempted to recruit a sample that was as broad and diverse as possible, including participants from different regions and demographics. Second, as pre-war data were not available, we were forced to rely on versions of existing mental health measures which were modified to capture change rather than absolute levels of symptoms. Although we cannot independently verify the validity of these measures of change, we selected measures that have been used in previous population studies in Ukraine, and both the factor structure and reliability were supported in the present analyses. Third, we relied on parent reports of change in child and adolescent mental health; therefore, our findings are likely subject to the well-documented low-to-moderate correspondence between parent and child reports of mental health problems [37]. Indeed, given that children tend to self-report greater levels of internalizing problems compared to parents [38], the increased problems observed in the present study may be an underestimate. Finally, although our measures were adapted to assess changes in mental health since the start of the war, the study design means we cannot infer causality, and rising levels of common mental health problems have been noted in countries not experiencing war in recent times [39].

### Implications and conclusions

Despite these limitations, this study provides important preliminary information on the early impact that the Russian invasion of Ukraine is having on child mental health, and following the growing humanitarian crisis and violence towards the civil population, it highlights an urgent need to develop a response. Brief trauma-informed psychological interventions may be useful in reducing trauma-related symptoms and building resilience in children [5, 40]; however, there is limited guidance on how to deliver mental health interventions in active conflict settings, and it is important that any such interventions are culturally sensitive [5]. As a starting point, there is a need to carefully assess the available mental health services in the country, and build capacity to address the needs of young people affected by the conflict [28]. Prior to the full-scale invasion in 2022, psychological therapies were poorly attended and resourced in Ukraine due to stigma [28]. Cooperation between the Ukrainian mental health-care community and international voluntary organizations, such as training for local practitioners, could be an important first step in addressing the mental health fallout of this war [5, 28].

In summary, we found evidence of increased parent reports of child internalizing, externalizing and attention problems in a diverse sample. Various individual, family, and war-related factors were associated with increased risks across the three domains. This study represents an early step in understanding how the Russian war has affected the mental health of children in Ukraine, and in identifying those requiring the most support in these incredibly trying times.

### Supplementary Information

Below is the link to the electronic supplementary material.

Supplementary file1 (DOCX 503 KB)

## Data Availability

Data are available from the corresponding author on reasonable request.
