# Supplementary material for: Change in child mental health during the Ukraine war: evidence from a large sample of parents
Source: Eur Child Adolesc Psychiatry. 2023 Jul 8;33(5):1495–502. doi: 10.1007/s00787-023-02255-z (PMC11098962; doi:10.1007/s00787-023-02255-z)
Supplement: Supplementary file 1 — Supplementary file1 (DOCX 503 KB) [file 787_2023_2255_MOESM1_ESM.docx]

**Change in child mental health during the Ukraine War: Evidence from parent-reports**

Supplementary files

Methods S1. Parent War trauma and Mental Health Measures

**Instructions**: In this section, we will ask you about different things you may have experienced during the war. We will then ask you about some reactions you may have had to these experiences.

Below are descriptions of events that you may have experienced following the Russian attack on Ukraine on February 24^th^, 2022. Please indicate if you experienced any of these events.

| 1. My home was damaged or destroyed. | No | Yes |
| --- | --- | --- |
| 1. Someone close to me had their home damaged or destroyed. | No | Yes |
| 1. I had to take shelter in an underground location. | No | Yes |
| 1. I witnessed the destruction of local infrastructure. | No | Yes |
| 1. I was displaced and had to move to another part of Ukraine. | No | Yes |
| 1. I was displaced and had to move to another country. | No | Yes |
| 1. My loved ones were displaced. | No | Yes |
| 1. I lost my job (temporarily or for an extended period). | No | Yes |
| 1. I experienced extreme financial hardship. | No | Yes |
| 1. I was unable to access necessities like food, water, electricity, or heating. | No | Yes |
| 1. I was unable to access essential healthcare like medicines or access to doctors. | No | Yes |
| 1. I was unable to sleep for prolonged periods of time. | No | Yes |
| 1. I heard air raid sirens. | No | Yes |
| 1. I heard or saw bombing or artillery fire. | No | Yes |
| 1. I heard or saw gun fire. | No | Yes |
| 1. I was stopped by military patrols. | No | Yes |
| 1. My hometown was occupied by invading Russian forces. | No | Yes |
| 1. I saw dead bodies of civilians. | No | Yes |
| 1. I saw dead bodies of Ukrainian soldiers. | No | Yes |
| 1. I saw dead bodies of Russian soldiers. | No | Yes |
| 1. I touched dead bodies (e.g., moved or buried dead bodies). | No | Yes |
| 1. Someone close to me died in the war. | No | Yes |
| 1. Someone close to me was physically hurt in the war. | No | Yes |
| 1. I was physically hurt in the war. | No | Yes |
| 1. I took part in defensive operations. | No | Yes |
| 1. I shot at the enemy forces. | No | Yes |
| 1. I was shot at by the enemy forces. | No | Yes |
| 1. I killed a member of the enemy forces. | No | Yes |
| 1. Is there any other event that you experienced that you would like to tell us about? | No | Yes |

Please give a brief description of this event: _________________

The following questions represent reactions people sometimes have following a very stressful life event. We would like to know if you have had any of these experiences **because of things you have experienced during the war.**

Please read each item carefully and indicate how much you have been bothered by each problem in the past month.

|  | ***Not at all*** | ***A little bit*** | ***Moderately*** | ***Quite a bit*** | ***Extremely*** |
| --- | --- | --- | --- | --- | --- |
| 1. Having upsetting dreams that replay part of the experience or are clearly related to the experience? | **0** | **1** | **2** | **3** | **4** |
| 2. Having powerful images or memories that sometimes come into your mind in which you feel the experience is happening again in the here and now? | **0** | **1** | **2** | **3** | **4** |
| 3. Avoiding internal reminders of the experience (for example, thoughts, feelings, or physical sensations)? | **0** | **1** | **2** | **3** | **4** |
| 4. Avoiding external reminders of the experience (for example, people, places, conversations, objects, activities, or situations)? | **0** | **1** | **2** | **3** | **4** |
| 5. Being “super-alert”, watchful, or on guard? | **0** | **1** | **2** | **3** | **4** |
| 6. Feeling jumpy or easily startled? | **0** | **1** | **2** | **3** | **4** |
| ***In the past month have the above problems:*** | | | | | |
| 7. Affected your relationships or social life? | 0 | 1 | 2 | 3 | 4 |
| 8. Affected your work or ability to work? | 0 | 1 | 2 | 3 | 4 |
| 9. Affected any other important part of your life such as parenting, or school or college work, or other important activities? | 0 | 1 | 2 | 3 | 4 |

Below is another set of problems that people sometimes experience after very stressful experiences. The questions refer to ways you typically feel, ways you typically think about yourself, and ways you typically relate to others.

**Again, we want to know if you have had any of these experiences because of things you have experienced during the war.**

Please read each statement and indicate how true is each statement of you?

|  | ***Not at all*** | ***A little bit*** | ***Moderately*** | ***Quite a bit*** | ***Extremely*** |
| --- | --- | --- | --- | --- | --- |
| 1. When I am upset, it takes me a long time to calm down. | 0 | 1 | 2 | 3 | 4 |
| 2. I feel numb or emotionally shut down. | 0 | 1 | 2 | 3 | 4 |
| 3. I feel like a failure. | 0 | 1 | 2 | 3 | 4 |
| 4. I feel worthless. | 0 | 1 | 2 | 3 | 4 |
| 5. I feel distant or cut off from people. | 0 | 1 | 2 | 3 | 4 |
| 6. I find it hard to stay emotionally close to people. | 0 | 1 | 2 | 3 | 4 |
| ***In the past month, have the above problems in emotions, in beliefs about yourself and in relationships:*** | | | | | |
| 7. Created concern or distress about your relationships or social life? | 0 | 1 | 2 | 3 | 4 |
| 8. Affected your work or ability to work? | 0 | 1 | 2 | 3 | 4 |
| 9. Affected any other important parts of your life such as parenting, or school or college work, or other important activities? | 0 | 1 | 2 | 3 | 4 |

| Table S1. Frequency and percentages of parent reported PSC-17 items. | |  |  |  |  |  |  |
| --- | --- | --- | --- | --- | --- | --- | --- |
|  |  | *Less often* | | *About the same* | | *More Often* | |
| Item | Content | N | % | N | % | N | % |
| PSC1 | Fidgety, unable to sit still | 149 | 12.04% | 871 | 70.36% | 218 | 17.61% |
| PSC2 | Feels sad, unhappy | 151 | 12.20% | 738 | 59.61% | 349 | 28.19% |
| PSC3 | Daydreams too much | 183 | 14.78% | 789 | 63.73% | 266 | 21.49% |
| PSC4 | Refuses to share | 211 | 17.04% | 841 | 67.93% | 186 | 15.02% |
| PSC5 | Does not understand other people’s feelings | 240 | 19.39% | 846 | 68.34% | 152 | 12.28% |
| PSC6 | Feels hopeless | 328 | 26.49% | 812 | 65.59% | 98 | 7.92% |
| PSC7 | Has trouble concentrating | 177 | 14.30% | 744 | 60.10% | 317 | 25.61% |
| PSC8 | Fights with other children | 361 | 29.16% | 773 | 62.44% | 104 | 8.40% |
| PSC9 | Is down on him or herself | 289 | 23.34% | 807 | 65.19% | 142 | 11.47% |
| PSC10 | Blames others for his or her troubles | 281 | 22.70% | 756 | 61.07% | 201 | 16.24% |
| PSC11 | Seems to be having less fun | 189 | 15.27% | 746 | 60.26% | 303 | 24.47% |
| PSC12 | Does not listen to rules | 180 | 14.54% | 826 | 66.72% | 232 | 18.74% |
| PSC13 | Acts as if driven by a motor | 238 | 19.22% | 818 | 66.07% | 182 | 14.70% |
| PSC14 | Teases others | 368 | 29.73% | 794 | 64.14% | 76 | 6.14% |
| PSC15 | Worries a lot | 158 | 12.76% | 635 | 51.29% | 445 | 35.95% |
| PSC16 | Takes things that do not belong to him or her | 396 | 31.99% | 763 | 61.63% | 79 | 6.38% |
| PSC17 | Distracted easily | 135 | 10.90% | 780 | 63.00% | 323 | 26.09% |


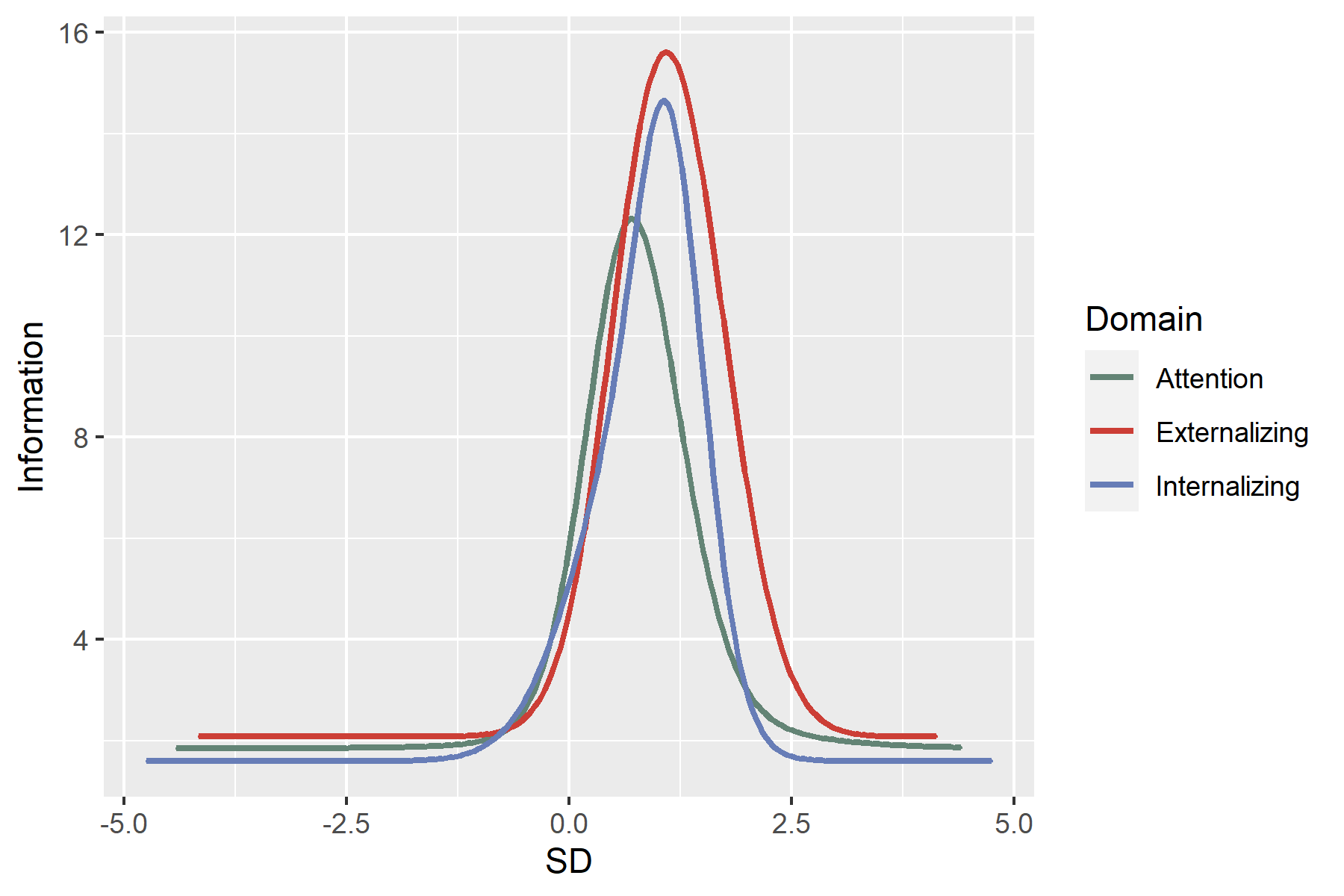


Figure S1. Total information functions for the three factors of the modified version of the PSC-17


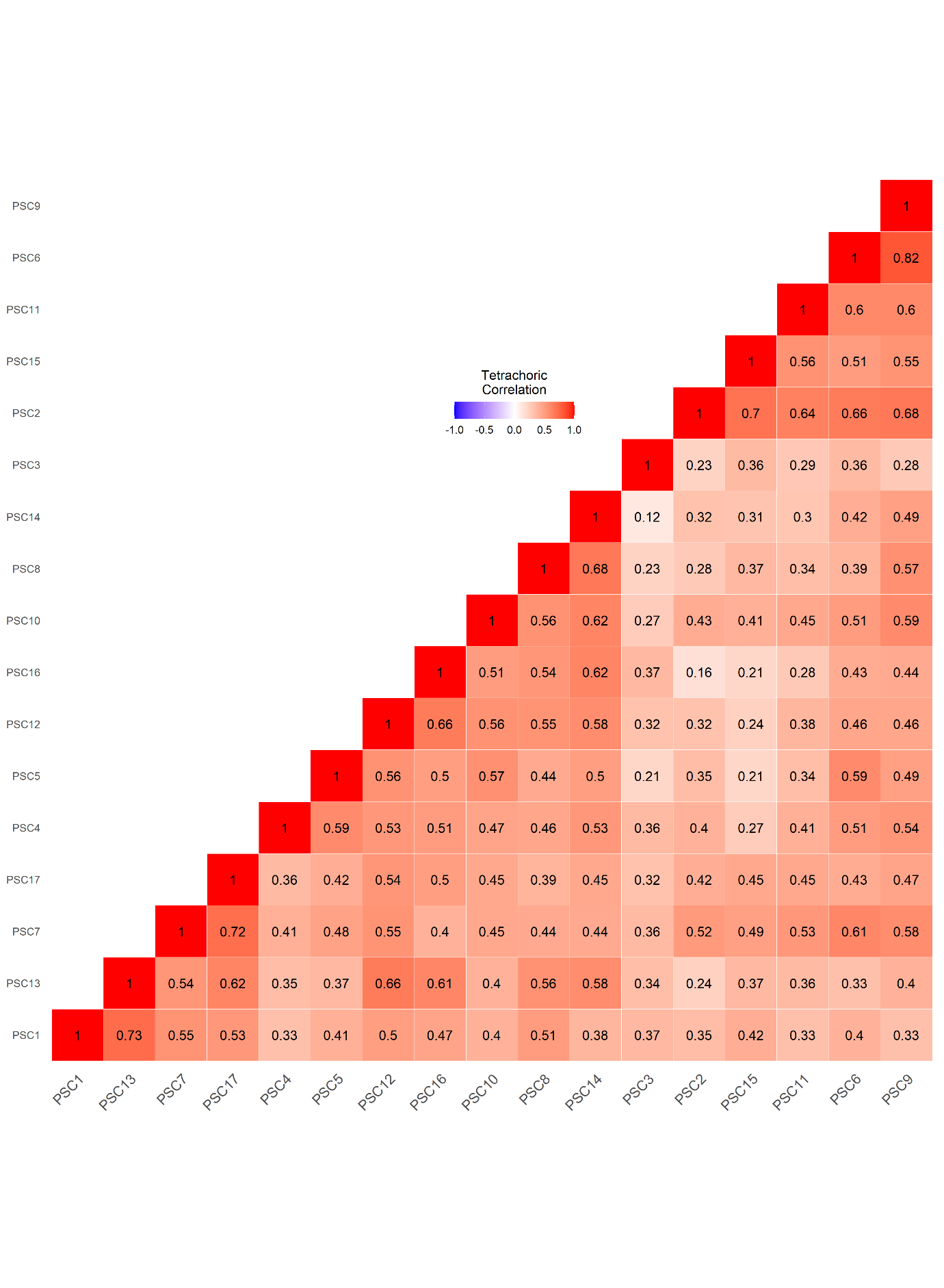


Figure S2. Tetrachoric correlation coefficients of the increases in PSC

| Table S1. Standardised factor loadings from the 3-factor CFA model of the modified PSC-17 | | | |
| --- | --- | --- | --- |
|  | Internalizing | Externalizing | Attention |
| PSC2 | 0.79 |  |  |
| PSC6 | 0.88 |  |  |
| PSC9 | 0.90 |  |  |
| PSC11 | 0.75 |  |  |
| PSC15 | 0.74 |  |  |
| PSC4 |  | 0.69 |  |
| PSC5 |  | 0.71 |  |
| PSC8 |  | 0.74 |  |
| PSC10 |  | 0.76 |  |
| PSC12 |  | 0.80 |  |
| PSC14 |  | 0.76 |  |
| PSC16 |  | 0.74 |  |
| PSC1 |  |  | 0.73 |
| PSC3 |  |  | 0.48 |
| PSC7 |  |  | 0.84 |
| PSC13 |  |  | 0.80 |
| PSC17 |  |  | 0.79 |
